# *Dermacentor reticulatus* (Fabricius, 1794) in Southwestern Poland: Changes in Range and Local Scale Updates

**DOI:** 10.3390/insects16090935

**Published:** 2025-09-05

**Authors:** Dorota Kiewra, Hanna Ojrzyńska, Aleksandra Czułowska, Dagmara Dyczko, Piotr Jawień, Kinga Plewa-Tutaj

**Affiliations:** 1 Department of Microbial Ecology and Acaroentomology, University of Wroclaw, Przybyszewskiego Str. 63, 51-148 Wroclaw, Poland; aleksandra.czulowska@uwr.edu.pl (A.C.); dagmara.dyczko2@uwr.edu.pl (D.D.); piotr.jawien@uwr.edu.pl (P.J.); kinga.plewa-tutaj@uwr.edu.pl (K.P.-T.); 2 Department of Climatology and Atmosphere Protection, Institute of Geography and Regional Development, University of Wroclaw, Pl. Uniwersytecki 1, 50-137 Wroclaw, Poland; hanna.ojrzynska@uwr.edu.pl

**Keywords:** *Dermacentor reticulatus*, ticks, expansion, SW Poland

## Abstract

The ornate dog tick *Dermacentor reticulatus* is a key vector of several pathogens and has been expanding its range across Europe, raising concerns about the associated veterinary and public health risks. This study aimed to assess the current distribution and local-scale expansion of *D. reticulatus* in southwestern Poland, particularly in and around the city of Wrocław. In 2024, host-seeking ticks were collected using the flagging method at 80 sites, including 30 previously monitored locations and 50 newly designated ones, selected based on land cover analysis and field verification. Spatial statistics and kriging method were applied to evaluate changes in the tick’s range compared to data from 2014–2019. The presence of *D. reticulatus* was confirmed at 68 sites, including 13 located beyond the previously estimated range. A shift in the mean center of tick occurrence toward the southeast was observed, along with an increase in the compact area of occurrence. The results indicate a continued expansion of *D. reticulatus* in the region, with urbanization and landscape structure likely influencing its spread. These findings underscore the importance of local-scale surveillance and spatial modeling in assessing the risk of tick-borne diseases.

## Simple Summary

The ornate dog tick *Dermacentor reticulatus* is a medically and veterinary important species whose distribution in Europe has significantly expanded in recent decades. In this study, we present updated data on the local-scale spread of *D. reticulatus* in southwestern Poland, based on systematic field monitoring. Our findings reveal a shift in tick occurrence toward the southeast, along the Odra River valley, accompanied by an increase in the compact area of occurrence. These results confirm the continued expansion of *D. reticulatus*, provide new insights into the dynamics of its colonization in previously unoccupied areas, and highlight the importance of local-scale surveillance for assessing epidemiological risk.

## 1. Introduction

The ornate dog tick *Dermacentor reticulatus*, an important vector for various pathogens, including *Babesia canis*, causing canine babesiosis, spotted fever group *rickettsia*, and tick-borne encephalitis virus, occurs in Eurasia from the Atlantic coast of Portugal to Western Siberia in regions with a generally mild climate [1,2]. Within its range, the occurrence of *D. reticulatus* is patchy as illustrated by published distribution maps [1,2,3]. However, the remarkable adaptability of *D. reticulatus* enables it to settle in new areas. This is evidenced by recent data on the significant expansion of these tick species across central and northeastern Europe [1,4]. In Germany, over the past 30 years, *D. reticulatus* spread has been observed from the southern regions to cover large areas of the country [5,6]. In Poland, two populations of *D. reticulatus* exist—Eastern and Western—with a previously *Dermacentor*-free zone in between that has been colonized since the 1990s [7,8,9]. Several studies conducted in Poland have documented the dynamic expansion of *D. reticulatus*, particularly in central and western regions of the country. These investigations were motivated by growing public health concerns related to tick-borne diseases and the need to monitor the distribution of vectors. The findings highlighted a significant increase in tick populations in previously non-endemic areas, suggesting environmental and climatic factors as key drivers. Interestingly, temperature and vegetation phenology do not appear to be significant factors in the spread of ticks [7]. Instead, according to a large-scale study conducted by Mierzejewska et al. [7] across most Polish voivodeships, the spread of *D. reticulatus* has been strongly linked to loss and fragmentation of forest areas, particularly near water sources, with colonized areas experiencing deforestation at twice the rate of non-colonized regions. Additionally, Karbowiak et al. [8] suggest that this expansion may be influenced by changes in average summer and winter temperatures in Europe, as well as shifts in the distribution and abundance of key mammalian hosts—such as red deer, elk, raccoon dogs, and foxes. Other factors contributing to the expansion of *D. reticulatus* include socio-economic factors, changes in ecosystem management, land use modifications, an increase in adult tick hosts, and the import of ticks through travel and trade [1,8]. These studies have been instrumental in shaping future tick management strategies by identifying environmental factors that influence tick distribution, thereby enabling the identification of high-risk areas for colonization.

In Lower Silesia, southwestern Poland, new outbreaks of *D. reticulatus* have been noted since the first decade of the 21st century [10,11]. Since then, many new locations in the north-western part of Lower Silesia have been found. A five-year systematic observation conducted in Wroclaw and its surroundings has demonstrated that the *D. reticulatus* range has expanded eastward at an estimated rate of 0.6–2.3 km/year [12]. Similar expansions have been observed in Slovakia, with the tick’s range extending 200 km northward and 300 m higher in elevation since earlier observations conducted in the 1950s and the 1970s [13].

Shifts in the distribution range of *D. reticulatus*, which heighten the medical and veterinary risks posed by tick-borne diseases, necessitate enhanced surveillance and monitoring of their current occurrence. The assessment of the spread of distributional changes encompasses large-scale modeling based on accurate species occurrence data using geostatistical and spatial analysis [4]. Although this provides valuable information for global-scale studies, the resolution of the map is too low for regional epidemiological purposes, underscoring the need for local-scale field monitoring to assess risk in a specific area [1]. The objectives of the study were: 1. to estimate the changes in the distribution range of *D. reticulatus* over a period of 5 years on a local scale in southwestern Poland; 2. to verify the previously estimated rate of spread of *D. reticulatus*.

## 2. Materials and Methods

### 2.1. Tick Collection and Study Area

**Figure 1:**
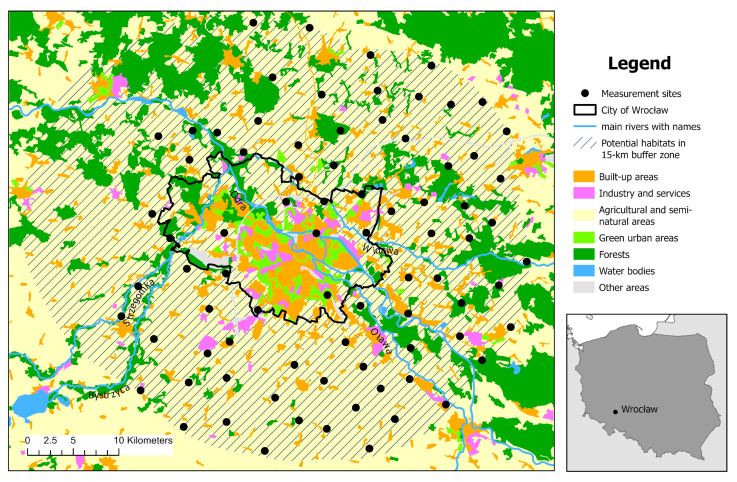
Sampling sites designated for *Dermacentor reticulatus* collection set against the land cover map of Wroclaw and its surroundings, SW Poland.

Ticks were collected from vegetation in spring (March–April) and/or in autumn (from the end of September to early November) 2024 with the flagging method at 80 sites located in Wroclaw and its surroundings, SW Poland (Figure 1). If a monitored site was classified as positive (tick presence) in spring, it was automatically no longer monitored in autumn. The remaining pool of monitoring sites was examined again in autumn. If a tick was recorded during these observations, these sites, along with the positive points from spring measurements, were treated as tick occurrence sites for that year.

The study sites included 30 sites designated in previous studies, which were monitored from 2014 to 2019 [12], as well as an additional 50 sites designated in 2024. Of these, 20 were monitored in spring and/or autumn 2024 and were located within an additional 5 km buffer zone surrounding the previously established area, while the remaining 30 sites were monitored only in autumn 2024. The expansion of the study area by an additional 30 sites located in the enlarged zone was driven by spring research results, which indicated an extension of the range beyond the predicted boundary [14]. All 50 new sites were designated within an additional 10 km buffer, excluding the western direction. This exclusion was based on probability lines established from 2014 to 2019, which indicated that the range boundary did not extend westward. The new sites were designated based on criteria previously described by Kiewra et al. [12,15], utilizing land cover maps, GIS analysis, and field site verification. The Odra River was treated as a potential barrier in the process of tick spread; therefore, new monitoring points were designated separately for areas located on the left and right banks of the Odra River. The 10 km buffer around the previously monitored areas exceeded the Urban Atlas 2018 land cover database for the Wrocław agglomeration, which was originally used to determine the potential range of habitats. For this reason, in this work, these agricultural and semi-natural areas were designated based on the CORINE Land Cover 2018, European Union’s Copernicus Land Monitoring Service information database [16]. This database has lower spatial resolution, and the Wrocław city surroundings quickly change, which is why on-site inspection of the proposed monitoring sites was necessary. New monitoring sites were designated randomly using the Create Random Points tool from the Sampling toolset in ArcGIS 10.7.1, assuming a minimum distance of 3 km between them.

Host-seeking ticks were gathered at least once annually at each site during the peak activity periods of *D. reticulatus*, i.e., in spring and/or in autumn. All collected ticks were placed in a container and transported to the laboratory, where they were stored in a refrigerator for a maximum of 1–2 days before being identified to species. The species classification of all tick specimens was determined based on morphology according to tick identification keys under a stereomicroscope [17,18]. If one or more *D. reticulatus* specimens were captured during a one-hour flagging in spring, the site was deemed positive for that year, and no autumn collection was conducted. Conversely, if no *D. reticulatus* ticks were found in spring, the one-hour flagging was repeated in autumn. A site was classified as negative if no *D. reticulatus* ticks were collected in both spring and autumn (for the 30 later established sites, only in autumn).

### 2.2. Analysis of D. reticulatus Tick Distribution and the Rate of Change in Its Range

For all monitored locations, separately for places with and without ticks, certain measures of spatial statistics were calculated using dedicated tools in ArcGIS 10.7.1 software: the Mean Center (MC), the Standard Distance (SD), and the Standard Deviation Ellipse (SDE). The Mean Center represents the average x and y coordinates of all locations classified as positive/negative for *D. reticulatus* presence. SD measures the degree to which tick occurrence/lack of tick occurrence points are concentrated or dispersed around this MC. The SDE measure, in addition to indicating the standard deviation of x and y coordinates of locations classified as positive/negative from MC, also quantitatively describes the orientation of their distribution. The orientation is determined by rotating the ellipse along the axis measured clockwise from north [19]. All mentioned measures were calculated for 2 different spatial ranges—the full area monitored in 2024 and the area representative of the 2019 measurements.

Due to the possibility of recording information on the occurrence of a tick in the monitoring locations in a binary way (0—no tick; 1—tick present), a simple nonparametric interpolation technique in the form of indicator kriging [20] was used to prepare maps of its probable range. For this purpose, the Geostatistical Analyst extension of the ArcGIS system was used. The theoretical variogram model was fitted to the experimental model using a semi-automatic procedure using the Gaussian function and the nugget effect. The model was evaluated using cross-validation. The calculated probability was presented in the form of raster maps with a range of values from 0 to 1, with an isoline of 100% probability of tick occurrence. The map area was limited to the spatial range of measurement sites to avoid inferences based on data extrapolated beyond the spatial range of observations.

Based on the results of monitoring from 2014–2019 and the estimated rate of spread of the tick [12], a line of its potential range in 2024 was determined. By comparing it with the map of the probability of tick occurrence in 2024, the differences in the rate of spread of the tick were estimated in various directional sectors: N, NE, E, SE, and S designated from the average center of tick occurrence in 2019. In places of greatest differences, a detailed analysis of land cover was carried out in terms of the possible occurrence of barriers to the spread of ticks.

## 3. Results

**Figure 2:**
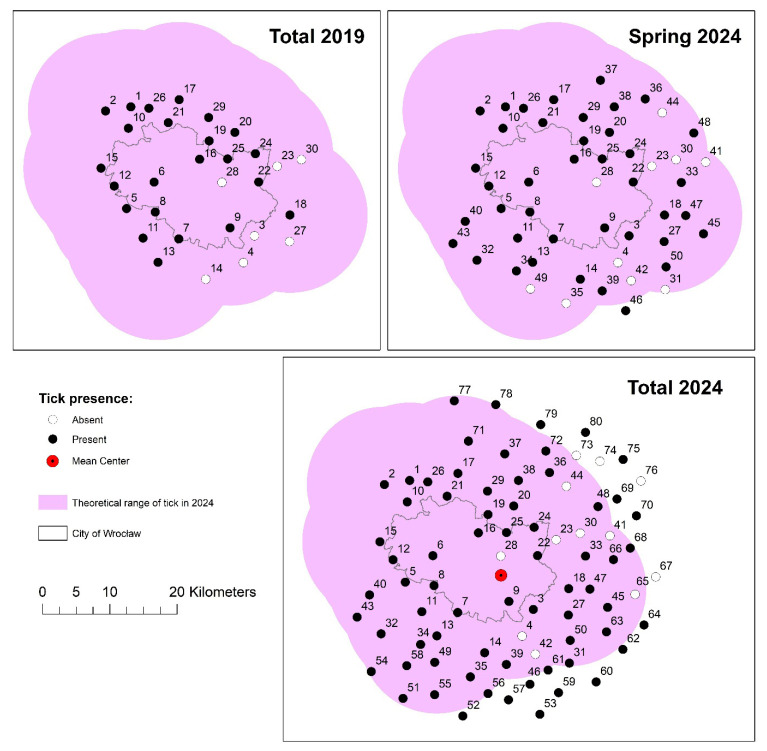
The presence of *Dermacentor reticulatus* in 2019 (30 sites), in spring 2024 (50 sites), and in total in 2024 (80 sites), against the backdrop of the theoretically estimated occurrence range in the Wrocław area and its surroundings.

The presence of *D. reticulatus* (Supplementary Figure S1) was confirmed in 68 out of the 80 sites surveyed (Figure 2). Positive positions were located on both sides of the Oder River. In comparison to the year 2019, when 23 out of 30 sites were identified as positive (i.e., at least one *D. reticulatus* specimen was found during a one-hour collection), three additional new sites of *D. reticulatus* occurrence were discovered. Moreover, among the additional 20 sites monitored in spring, 15 were found to be positive. These sites were located both within the theoretically designated range based on studies conducted from 2014 to 2019 and one (site 46) beyond the range (Figure 2). In autumn, ticks were additionally recorded at the next 2 sites in new 5 km buffer zones monitored earlier in spring, as well as at 25 of the 30 newly surveyed locations. In total, during 2024, the presence of *D. reticulatus* was confirmed at 13 sites located beyond the theoretically estimated range. The sites assessed as negative in 2024 were located both within the monitored area, surrounding positive sites, and outside the area towards the east.

The average tick occurrence center (MC) in 2024 was located in the eastern part of Wrocław, slightly south of monitoring point no. 28. The compact area of occurrence extended beyond the city limits at a distance of SD equal to 17.3 km (1 standard deviation). The identified compact tick-free areas were distributed in an island-like manner, and were spread on the NNE-SSW axis, at a deviation distance SDE of 8.1 to 14 km, mainly in the north-eastern, but also south-eastern suburbs of Wrocław. Among the monitoring sites located within the city limits, ticks were not found at site no. 28. This point, located on the Odra embankments, was characterized by a very high degree of urbanization of the surrounding areas, as it was located closer than 50 m to the nearest buildings, and the number of inhabitants in the area of the nearest 1 km^2^ reached almost 3700. In the history of measurements, the occurrence of a tick was recorded only once, in 2017. Since then, the surroundings have been subject to even stronger anthropogenic pressure. For this reason, this point was not included as a potential habitat in further analysis.

**Figure 3:**
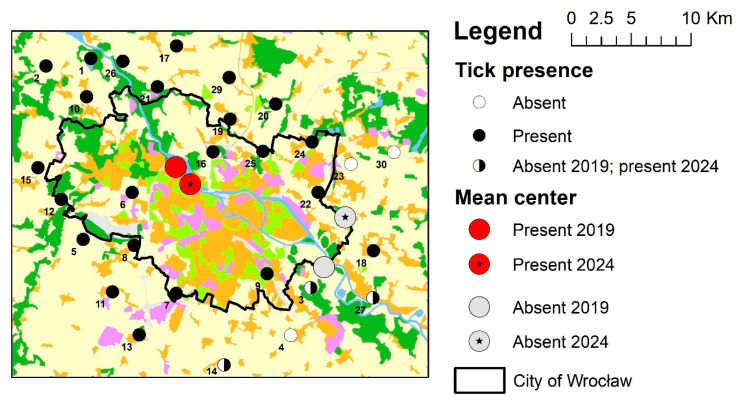
The mean center of *D. reticulatus* presence area.

Based on the analysis of 30 sites monitored both in 2019 and 2024, a noticeable shift in the Mean Center of *D. reticulatus* occurrence was observed, moving in a south-easterly direction, along the course of the Odra Valley (Figure 3). A shift was also observed in the Mean Center of tick absence, which moved in a north-easterly direction. The compact tick occurrence area increased from 10.5 km to 11.4 km (1 standard deviation), and a deviation of this area towards the south was observed (difference in deviation 16 degrees). In 2019, the compact tick-free area was strongly elongated in the NNE-SSW axis, with the distance of SDE from 4.4 to 13.6 km. In 2024, a very small representation of negative points was noted with an island effect of their occurrence, therefore it was not possible to calculate their SD and SDE.

Among the sites where the presence of *D. reticulatus* was not recorded in 2024, most were characterized by the close proximity of development (<50 m) of a suburban or agricultural nature with a moderate number of people in the vicinity of 1 km^2^ (<280 inhabitants) and a significant share (50–90%) of the area of potential habitats within a radius of 1 km (Supplementary Table S1). It should be added that such characteristics of the surroundings also concerned the monitoring points marked as positive. The difference between these positions, however, concerned the intensity of development in specific directions. In the case of certain negative sampling sites, the absence of tick development in specific directional sectors may have been influenced by the presence of disruptive industrial facilities, such as industrial plants (site no. 23, 30, 65, 67), large-scale horticultural enterprises—greenhouses (sites no. 4, 42), or the proximity of an expressway (sites no. 44, 76).

**Figure 4:**
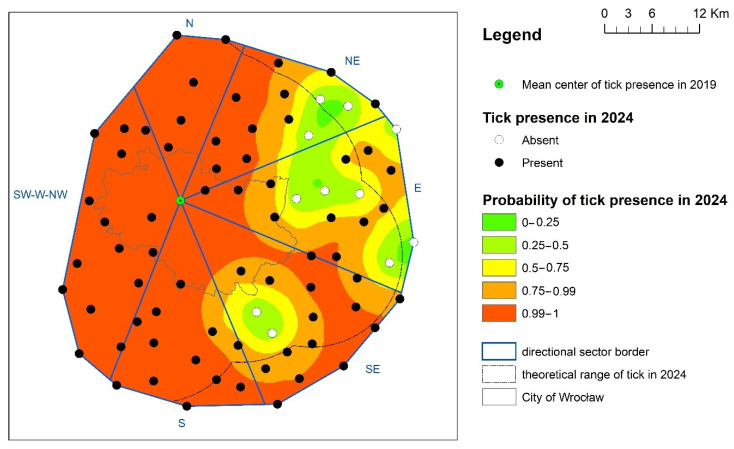
The presence and the probability of *D. reticulatus* presence in directional sectors in Wrocław and its surroundings in the year 2024.

The analysis of the probability map of *D. reticulatus* occurrence in relation to previous observations, and above all the estimated rate of spread of the tick since 2019, showed a varied rate of its expansion (Figure 4). In the northern and southern directional sectors, in the entire area of its predicted occurrence, as well as outside it, the presence of the tick was recorded and in most of the area a 100% probability of its occurrence was estimated (Table 1). In the south-eastern sector, in the area of potential tick occurrence, island negative positions were recorded, while the range of positive positions also reached beyond the area of its potential occurrence. However, in most of the areas in the analyzed sector, a high (>75%) probability of *D.reticulatus* occurrence prevailed. A similar situation was also recorded in the NE sector; however, in this direction outside the area of potential tick occurrence, the probability of its encounter decreased much faster (Figure 4, Table 1). The relatively lowest probability of *D.reticulatus* occurrence was noted for the eastern sector; however, even here, positive sites were noted outside the estimated area of its potential occurrence. The identified islands of tick absence in the SE and E sectors were separated by a tick expansion corridor, which aligns with the course of the Odra Valley and heavily built-up agricultural areas south of Wrocław.

## 4. Discussion

The conducted studies provide evidence that the geographical distribution of *Dermacentor reticulatus* has been steadily expanding. Numerous studies conducted across Europe provide compelling evidence for the ongoing expansion of *D. reticulatus*. This trend has been documented in various countries, including Poland, Germany, the Czech Republic, Slovakia, and the Baltic states, where the tick has been observed in previously unrecorded areas [6,7,11,21]. The growing number of confirmed localities, often supported by long-term monitoring and spatial modeling, highlights the dynamic nature of the species’ range. These findings suggest that *D. reticulatus* is not only expanding its distribution but also adapting to a broader range of environmental conditions, including anthropogenically altered landscapes.

Our research has led to the identification of several new localities where *D. reticulatus* is present. These findings not only confirm the ongoing expansion of the species but also contribute to refining the current understanding of its geographical distribution. The newly detected sites, some of which were previously considered unsuitable or unoccupied, highlight the dynamic nature of the tick’s range and underscore the importance of continuous monitoring efforts. It is important to emphasize that the tick’s expansion rate, as estimated from studies carried out between 2014 and 2019 [12], has proven to be both reliable and representative. Although, due to the enlarged study area, we cannot directly compare the results related to the rate of tick movement, the analysis in the originally assumed area shows a shift towards the southeast. The rate of spread may be influenced by additional factors, including changes in land use and host population dynamics, which accelerate or slow down the expansion, contributing to the formation of an “island effect”. Compared to 2019, the presence of *D. reticulatus* was confirmed at several new locations, including three sites that were negative in 2019 and an additional 42 out of 50 newly designated sites. Moreover, *D. reticulatus*-positive sites were documented both within the theoretically designated range (based on studies from 2014 to 2019) and beyond the expected distribution limits. It is also noteworthy that some locations within the theoretical range were found to be tick-free, although of these negative sites were surrounded by positive ones. This suggests that the observed tick-free patches are likely to come under increasing pressure, which may eventually lead to their disappearance. Consequently, the boundary of the tick’s distribution is likely to shift further, adopting a more meridional orientation.

In our study, both positive and negative sampling sites in close proximity to urban or rural built-up areas. These locations typically exhibited moderate population density within a 1 km^2^ radius. Furthermore, a significant portion of the surrounding landscape within this radius consisted of habitats potentially suitable for *D. reticulatus*. However, the absence of ticks at certain sites may have been influenced by the presence of disruptive industrial infrastructure, such as factories, large-scale horticultural enterprises, or nearby expressways. These anthropogenic factors may contribute to habitat degradation, including impacts on host species, thereby reducing the suitability of the environment for sustaining tick populations. Many authors have noted that anthropogenic pressures—including artificial light and roadway infrastructure—can alter the behavior, physiology, and survival of vertebrates [22,23,24,25]. In the case of the large greenhouse complex in Siechnice (less than 3 km from sites no. 4 and 42, which form a negative island in the SE sector), the influence of ecological pollution from artificial light cannot be ruled out. This effect was previously observed in studies on bird activity in the area [26]. Undoubtedly, the emergence of *D. reticulatus*-free patches within the species’ established range warrants further investigation. Understanding the ecological, environmental, and anthropogenic factors contributing to the formation and persistence of these negative islands may provide valuable insights into the mechanisms regulating tick distribution.

On the other hand, the presence of ecological corridors may facilitate or accelerate the species’ expansion by enabling easier dispersal of tick hosts across the landscape. In the Wrocław area, the most functionally important elements of natural connectivity are the ecological corridors associated with river valleys—particularly those of the Odra, Bystrzyca, Oława, and Widawa rivers [27]. Our research indicates that the Odra Valley appears to be a key factor contributing to the rapid southeastward expansion of the tick, with positive sites inhabited by *D. reticulatus* located on both sides of the Oder River. These findings may contribute to future tick research by highlighting the importance of landscape features and ecological corridors in shaping tick distribution. Understanding such mechanisms can support the development of more targeted tick monitoring and management strategies, especially in areas at risk of colonization. Ecological corridors play a crucial role in connecting isolated areas with elements of natural ecosystems. River valleys, which not only provide favorable habitats for *D. reticulatus* but also serve as migration pathways for wildlife and the pathogens they carry, are among the most prominent examples [8]. In the Odra River Valley, several mammal species may play a role in supporting local tick populations and facilitating their expansion. Notable examples include the red fox (*Vulpes vulpes*), wild boar (*Sus scrofa*), and roe deer (*Capreolus capreolus*), all of which are common in the region and known to serve as hosts for *D. reticulatus*. Their presence in riparian and forested habitats may contribute to the observed spread of this tick species. The role of river valleys as key habitats in the spread of *D. reticulatus* has also been confirmed by studies conducted along the Wieprz River Valley in eastern Poland [28], which demonstrated that habitats within riverine ecological corridors can be considered preferred environments for this species.

The findings of this study confirm the continued expansion of *D. reticulatus* and reveal the presence of both newly colonized areas and unexpected tick-free patches within the species’ theoretical range. These results underscore the complex interplay of environmental, ecological, and anthropogenic factors shaping the current distribution of the species. Future studies should focus on identifying potential barriers to colonization, assessing habitat quality, and evaluating the role of host availability and landscape fragmentation in shaping these localized absences. A better understanding of these mechanisms will be essential for predicting future changes in the tick’s range and for developing effective monitoring and control strategies, particularly in the context of vector-borne disease risk.

## 5. Conclusions

Our research indicates a clear shift in the *D. reticulatus* population, particularly a rapid southeastward expansion, with the Odra Valley acting as a key ecological corridor facilitating this movement. We conclude this shift based on comparative data from previous studies and current field observations, which show new localities of tick presence. This shift may have a significant impact on vector capacity and pathogen transmission in Poland. As *D. reticulatus* expands into new areas, it may encounter different host communities and environmental conditions, potentially altering its role in the transmission of pathogens such as *Babesia canis* or *Rickettsia* spp. In terms of animal and human health, the expansion of this tick species could lead to increased risk of tick-borne diseases and the need for more intensive monitoring and prevention strategies.

## Figures and Tables

**Table 1:** Percentage of areas with *D.reticulatus* presence probability class in directional sectors.

	0.99–1	0.75–0.99	0.5–0.75	0.25–0.5	0–0.25
N	100	0	0	0	0
NE	45.3	20.4	14.2	16.8	3.4
E	10.9	34.4	29.7	23.2	1.8
SE	44.7	36.9	12.2	6.2	0
S	87.7	11.6	0.7	0	0

## Data Availability

The original contributions presented in this study are included in the article. Further inquiries can be directed at the corresponding author.

## Supplementary Materials

The following supporting information can be downloaded at: https://www.mdpi.com/article/10.3390/insects16090935/s1, Figure S1: Adult *Dermacentor reticulatus*: (A) female, (B) male. Table S1. Location and selected characteristics of all flagging sites in 2024 in Wroclaw and its surroundings, Lower Silesia, Poland (sites where the presence of *D. reticulatus* was not recorded are marked in gray).
