# Supplementary material for: Dermacentor reticulatus (Fabricius, 1794) in Southwestern Poland: Changes in Range and Local Scale Updates
Source: Insects. 2025 Sep 5;16(9):935. doi: 10.3390/insects16090935 (PMC12471085; doi:10.3390/insects16090935)
Supplement: Supplementary file 1 [file insects-16-00935-s001.zip › insects-3749524-Table S1.pdf]

Table S1. Location and selected characteristics of all flagging sites in 2024 in Wroclaw and its surroundings, Lower Silesia, Poland (sites where the presence of *D. reticulatus* was not recorded are marked in gray).

No. of the Site	Longitude [°]	Latitude [°]	Potential Habitats in 1-km Buffer Zone [%]	Distance Class to the Nearest Built-up Area [m]	Type of the Nearest Built-Up Area [m]	Population in 1 km x 1 km Grid
1	16,858	51,230	36,5	200-500	rural	0
2	16,805	51,224	99,7	200-500	rural	79
3	17,128	51,062	58,9	<50	rural	24
4	17,106	51,026	67,8	<50	rural/suburban	258
5	16,855	51,094	94,8	>500	rural	103
6	16,912	51,130	75,5	<50	urban	1203
7	16,968	51,055	52,0	<50	uban	1194
8	16,916	51,090	65,8	<50	suburban	1640
9	17,076	51,072	0,6	<50	suburban	4016
10	16,854	51,201	79,0	<50	suburban	110
11	16,892	51,055	70,4	<50	rural	175
12	16,828	51,124	59,7	100-200	suburban	7
13	16,925	51,023	54,5	<50	rural	337
14	17,028	51,002	90,8	<50	rural	21
15	16,799	51,147	96,6	<50	suburban	25
16	17,008	51,163	42,2	<50	suburban	51
17	16,960	51,241	93,9	<50	rural	51
18	17,202	51,091	55,3	<50	rural	692
19	17,027	51,187	59,3	<50	suburban	675
20	17,081	51,200	76,2	200-500	rural	0
21	16,939	51,210	54,5	<50	rural	309
22	17,134	51,134	70,4	<50	urban	1626
23	17,173	51,156	79,8	<50	suburban	144
24	17,126	51,172	32,4	<50	suburban	53
25	17,067	51,164	69,9	<50	urban	0
26	16,897	51,229	76,6	200-500	rural	0
27	17,203	51,056	35,6	<50	rural	341
28	17,056	51,132	0,0	<50	urban	3675
29	17,024	51,219	78,8	<50	rural	358
30	17,224	51,166	68,5	<50	rural	286
31	17,208	50,991	78,0	<50	rural	363
32	16,807	51,024	100,0	<50	rural	0
33	17,236	51,135	99,9	<50	rural	3
34	16,891	51,011	74,9	<50	suburban	0
35	16,999	50,970	93,1	<50	rural	166
36	17,155	51,246	92,2	<50	rural	0
37	17,059	51,269	83,8	<50	rural	240
38	17,090	51,234	30,1	<50	rural	3
39	17,074	50,988	87,0	<50	rural	229
40	16,780	51,075	95,0	<50	rural	40
41	17,287	51,163	47,9	<50	rural	111
42	17,135	51,002	91,4	<50	rural	265
43	16,755	51,045	59,9	<50	rural	141
44	17,192	51,228	90,6	<50	rural	85
45	17,286	51,067	20,0	<50	rural	4
46	17,125	50,962	100,0	>500	rural	15
47	17,248	51,091	62,4	<50	rural/suburban	247
48	17,261	51,202	47,4	<50	rural	0
49	16,922	50,988	93,4	<50	rural	74
50	17,208	51,022	54,1	<50	rural	41
51	16,858	50,938	94,3	<50	rural	7
52	16,985	50,917	100,0	200-500	rural	0
53	17,148	50,922	94,6	<50	rural	51
54	16,788	50,973	85,1	<50	rural	185
55	16,924	50,945	99,8	200-500	rural	3
56	17,037	50,948	90,2	<50	rural	1
57	17,081	50,940	88,1	<50	rural	112
58	16,863	50,982	87,2	<50	rural	0

59	17,186	50,952	100,0	100-200	rural	0
60	17,265	50,967	61,8	<50	rural/suburban	344
61	17,163	50,981	100,0	>500	rural	0
62	17,320	51,012	23,1	<50	rural	79
63	17,285	51,035	76,7	<50	rural/suburban	65
64	17,364	51,045	84,8	<50	rural	12
65	17,344	51,086	50,8	<50	rural	15
66	17,296	51,131	63,7	>500	rural	191
67	17,387	51,109	60,4	>500	rural	0
68	17,331	51,147	68,7	<50	rural	159
69	17,300	51,212	97,8	<50	rural	0
70	17,343	51,191	99,7	<50	rural	0
71	16,981	51,285	72,1	100-200	rural	0
72	17,147	51,274	78,9	<50	rural	23
73	17,212	51,269	91,3	<50	rural	60
74	17,262	51,262	91,6	>500	rural	9
75	17,312	51,266	100,0	200-500	rural	7
76	17,351	51,237	95,5	<50	rural/suburban	193
77	16,948	51,338	95,1	>500	rural	120
78	17,037	51,334	94,0	<50	rural	49
79	17,134	51,309	76,1	<50	rural	83
80	17,230	51,300	91,2	>500	rural	0
