# Supplementary figures and images for: Dermacentor reticulatus (Fabricius, 1794) in Southwestern Poland: Changes in Range and Local Scale Updates
Source: Insects. 2025 Sep 5;16(9):935. doi: 10.3390/insects16090935 (PMC12471085; doi:10.3390/insects16090935)

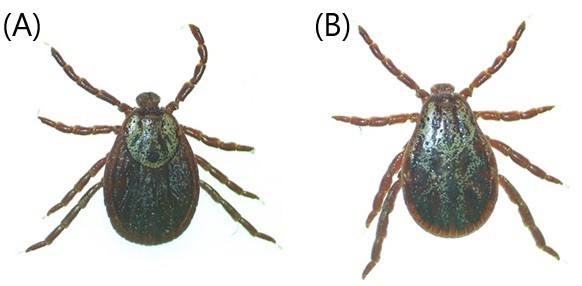

Supplement: Supplementary file 1 [file insects-16-00935-s001.zip › insects-3749524-Figure S1.jpg]
